# A light-controlled switch after dual targeting of proliferating tumor cells via the membrane receptor EGFR and the nuclear protein Ki-67

**DOI:** 10.1038/srep27032

**Published:** 2016-06-01

**Authors:** Sijia Wang, Gereon Hüttmann, Thomas Scholzen, Zhenxi Zhang, Alfred Vogel, Tayyaba Hasan, Ramtin Rahmanzadeh

**Affiliations:** 1 Key Laboratory of Biomedical Information Engineering of Ministry of Education, Institute of Biomedical Analytical Technology and Instrumentation, School of Life Science and Technology, Xi’an Jiaotong University, Xi’an 710049, P. R. China; 2 Institute of Biomedical Optics, University of Lübeck, Peter-Monnik-Weg 4, 23562 Lübeck, Germany; 3 Department of Immunology and Cell Biology, Research Center Borstel, Parkallee 22, 23845 Borstel, Germany; 4 Wellman Center for Photomedicine, Massachusetts General Hospital, Harvard Medical School, 50 Blossom St, Boston, MA 02114, USA

## Abstract

Using nanotechnology for optical manipulation of molecular processes in cells with high spatial and temporal precision promises new therapeutic options. Especially tumor therapy may profit as it requires a combination of both selectivity and an effective cell killing mechanism. Here we show a dual targeting approach for selective and efficient light-controlled killing of cells which are positive for *epidermal growth factor receptor* (EGFR) and Ki-67. Liposomes with the covalently linked EGFR antibody Erbitux enabled selective uptake of FITC-labeled Ki-67 antibody TuBB-9 in EGFR-positive cells pre-loaded with the photoactive dye BPD. After irradiation at 690 nm, BPD disrupted the endosomal membranes and delivered the antibodies to the nucleoli of the cells. The second irradiation at 490 nm activated the FITC-labeled TuBB-9, which caused inactivation of the Ki-67 protein and subsequent cell death via apoptosis. Efficient cell killing was possible at nanomolar concentrations of TuBB-9 due to the effective transport by immune liposomes and the high efficacy of the Ki-67 light-inactivation. Delivery of the liposomal constructs and cell destruction correlated well with the EGFR expression pattern of different cell lines (HeLa, OVCAR-5, MCF-7, and human fibroblasts), demonstrating an excellent selectivity.

Molecular targeted therapies are promising approaches for cancer treatment and an increasing number of drugs targeted to specific molecular pathways are approved for cancer therapy^1^. Many possible targeting agents block essential biochemical pathways or mutant proteins that are involved in tumor growth and cancer cell proliferation^2^. In comparison to traditional chemotherapies, the selective inactivation of cellular molecules causes much lower or even no systemic side effects. Due to their specific working mechanism molecular targeted therapies are also predestinated for personalized treatment^2^,^3^. However, the choice of the target molecule and the delivery of functional agents remain crucial challenges. Main hurdles are an effective and selective delivery into cancer cells, the cytoplasmic release of the delivered agents and an effective cellular destruction mechanism^4^.

In the present study, we designed a systematic strategy for a combined targeting of two target proteins, EGFR on the cell surface and Ki-67 in the cell nucleus (Fig. 1). Both proteins are critical prognostic indicators of disease stage and overexpressed in the target cells. The combined targeting can significantly enhance the selectivity. EGFR is overexpressed in many malignancies and is especially in ovarian cancer, colorectal cancer and head and neck cancer associated with poor prognosis^5^. Inhibition of EGFR activity with the monoclonal antibody Erbitux leads to reduced cell growth and reduced metastasis^6^,^7^,^8^. Erbitux is clinically approved for the treatment of metastatic colorectal cancer and metastatic head and neck cancer.

The nuclear protein Ki-67 proved to be an excellent target to trigger cell death after light inactivation with the antibody TuBB-9^9^,^10^. Ki-67 is strongly expressed in proliferating cells^11^,^12^ and is an established prognostic indicator for the assessment of cell proliferation in biopsies from cancer patients^13^. The monoclonal antibody TuBB-9 is the only Ki-67 antibody, which specifically recognizes a physiologically active form of Ki-67^14^. Covalently linked to the photoactive dye FITC, TuBB-9 effectively kills the cells after light irradiation.

The challenge of transfering the large TuBB-9-FITC conjugate into the cells was overcome by encapsulating the conjugate in an immune liposome with the antibody Erbitux on the liposome surface. Immune liposomes were synthesized from polyethylene glycol (PEG)-modified liposomes, which are extensively investigated as carriers for drugs and macromolecules with biological activities. PEG residues form an aqueous layer on the liposome surface which avoids their trapping in the reticuloendothelial system (RES)^15^,^16^. Therefore, PEG-modified liposomes have a long circulation time and tend to accumulate in tumor tissues through leaky angiogenic vessels, which is also known as enhanced permeability and retention (EPR) effect^17^,^18^. In addition, liposomes are able to reduce off-target toxicity of the encapsulated agents^19^ and PEG-modified liposomes can easily be conjugated with ligands on the liposome surface such as antibodies^20^,^21^,^22^, proteins^23^ and peptides^24^,^25^. With these modifications, liposomes are able to achieve more selective drug delivery to tumor cells. These ligands recognizing tumor or tumor secreted molecules can be conjugated to the PEG-chains on the liposome surface by introducing an active group to the head of the PEG-chains. Here we use the anti-EGFR antibody Erbitux conjugated to the liposome surface for cell selective delivery of TuBB-9-FITC conjugates. We linked a maleimide group on the head of the PEG-chains on the liposome surface to covalently bind Erbitux on the surface of TuBB-9-FITC-loaded liposomes for a preferred uptake of the conjugates by EGFR-positive cells.

However, liposomes, especially when encapsulating macromolecules like antibodies, are often finally degraded in lysosomes, before they can exert their action, because after uptake through endocytosis they enter the endosomal pathway^26^,^27^. Nanoconjugation of Erbitux can also alter the cellular uptake mechanism^28^,^29^. For the efficient cytoplasmic release of the TuBB-9-FITC conjugates, which is the prerequisite for the subsequent photo-triggered inactivation of the Ki-67 protein, we applied an established two-step light controlled approach introduced in our previous work^30^. In the first step irradiation at 690 nm was applied to trigger the disruption of liposome and endosomal membranes with the help of the pre-incubated photosensitizer BPD. This irradiation, also known as photochemical internalization (PCI)^31^, leads to liposome and endosome rupture and the release of TuBB-9-FITC into the cytoplasm. After 48 h TuBB-9-FITC locates in the nucleoli of the cells. In the second step light irradiation at 490 nm triggered the photoinactivation of Ki-67 by reactive oxygen species generated from FITC and led to subsequent cell death. Due to the specific transport by immune liposomes, light triggered endosomal release and the efficient photoinactivation of Ki-67, profound cell killing of EGFR-positive cancer cells was demonstrated even in nanomolar concentrations of TuBB-9-FITC.

## Results

### Characterization of non-functionalized and immune liposomes

**Figure 2:**
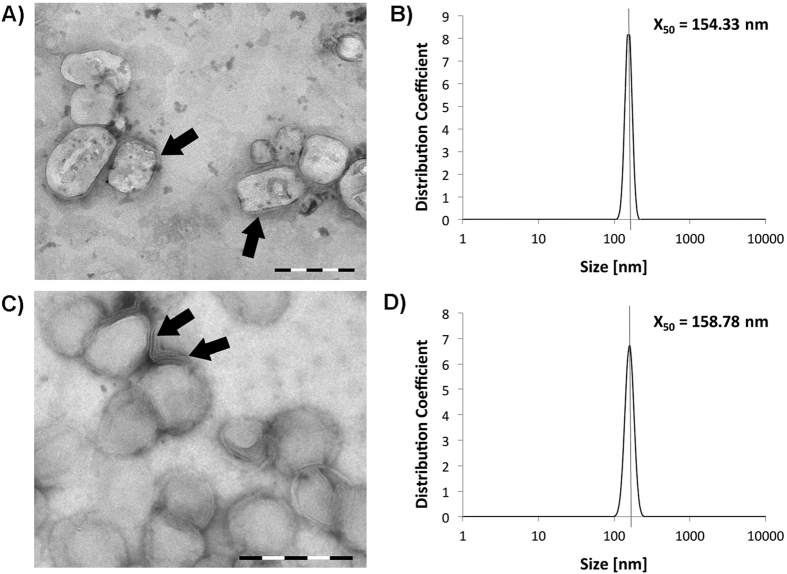
Characterization of immune liposomes (**A,B**) and non functionalized liposomes (**C,D**). Transmission electron microscopy images (TEM) showed the round shape and lipid bilayer structure (arrows) of the liposomes (**A,C**; size bars: 200 nm). Dynamic light scattering measurements (DLS) confirmed a median size for the liposomes of approximately 160 nm (**B,D**).

The sizes and shapes of the non-functionalized and antibody-functionalized liposomes were characterized by transmission electron microscopy (TEM). Both liposomal formulations encapsulated the TuBB-9-FITC antibody conjugates, while only the immune liposome had the EGFR antibody Erbitux on the surface. The liposomes showed spherical shape with a lipid bilayer membrane (Fig. 2A,C). The median size of the liposomes of 160 nm was confirmed by dynamic light scattering (DLS) measurements (Fig. 2B,D).

### Cellular uptake of TuBB-9-FITC with non-functionalized and immune liposomes

**Figure 3:**
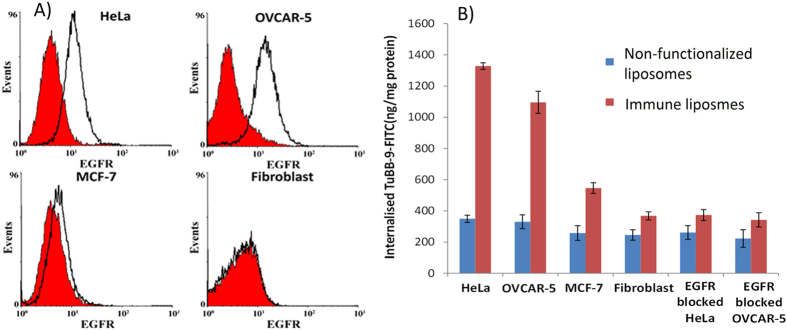
The expression level of EGFR as measured by flow cytometry in different cell lines (**A**) correlates with the cellular uptake of TuBB-9-FITC with immune liposomes (**B**). Blocking of EGF-receptor by free Erbitux before liposome incubation inhibited uptake via the EGF-receptor. EGFR expression levels were measured by flow cytometry. To estimate the cellular uptake efficiency, the fluorescence of TuBB-9-FITC was measured in lysed cells.

In order to test the cellular uptake efficiency of non-functionalized and immune liposomes, cell lines with different expression levels of EGFR on the membrane were used. The membrane expression levels of EGFR were confirmed by flow cytometry analysis (Fig. 3A). We used highly EGFR-expressing cell lines OVCAR-5 and HeLa, the low EGFR-expressing cell line MCF-7 and EGFR-negative human fibroblasts. The uptake was measured as fluorescence intensity of cell lysates after 4 h incubation with the conjugates. Fluorescence of lysed cells was compared with the fluorescence of known amounts of TuBB-9-FITC and the results were plotted as the amount of antibody per total protein mass (Fig. 3B). Non-functionalized liposomes showed nearly equal uptake of TuBB-9-FITC in all tested cell lines. For immune liposomes, the cellular uptake of TuBB-9-FITC was higher in the EGFR over-expressing cells OVCAR-5 and HeLa. Here, more than three times higher TuBB-9-FITC concentrations were measured with immune liposomes compared to bare liposomes. The low EGFR levels expressing cell line MCF-7 showed also a significant increase in TuBB-9-FITC delivery with immune liposomes. If the EGF-receptor on the cell membrane was blocked by prior incubation with 2 mg/ml free Erbitux antibody (last two samples of Fig. 3B), the uptake of the immune liposomes was nearly as low as the uptake by the EGFR-negative fibroblasts.

### PCI irradiation and nucleolar localization of TuBB-9-FITC

**Figure 4:**
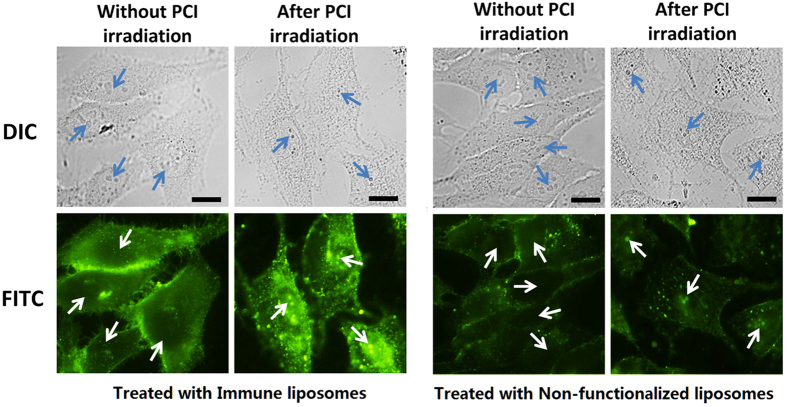
Intracellular localization of TuBB-9-FITC delivered by non-functionalized and immune liposomes without light irradiation and after 690 nm PCI irradiation for photochemical internalization (PCI). Images were taken with the same exposure time of 1 s. Arrows point to the nucleoli, size bars: 10 μm.

Fluorescence microscopy images showed that most of the internalized TuBB-9-FITC, delivered by liposomes, was trapped within endoplasmatic structures, probably endosomes (Fig. 4). For a release into the cytoplasm we used photochemical internalization (PCI). The entrapped TuBB-9-FITC was delivered to the cytosol by co-incubation of the cells with the photosensitizer BPD and irradiation at 690 nm. Fluorescence images of HeLa cells that were incubated with BPD for 18 h and TuBB-9-FITC for 4 h showed with both types of the liposomes fluorescence in the plasma membrane and the cytoplasm in certain dot like patterns (Fig. 4 without PCI irradiation). The fluorescence intensity was much higher for the immune liposomes. 48 h after irradiation with 18 mW/cm^2^ at 690 nm light for 30 s, the FITC fluorescence is localized inside the nucleoli of the cells (arrows in Fig. 4 after PCI irradiation). This relocalisation of TuBB-9-FITC from endosomal structures to the cell nucleus is shown in higher magnification in Supplementary Fig. S1. Relocalization was also observed in previous studies after cytosolic microinjection^9^ or after PCI^30^.

### Ki-67 inactivation after nuclear delivery of TuBB-9-FITC

**Figure 5:**
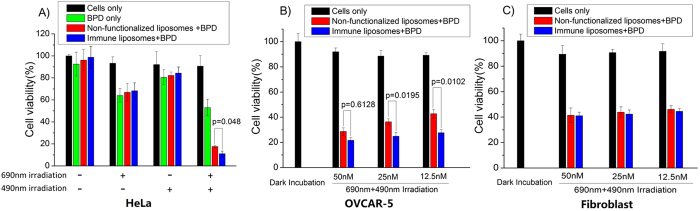
Cytotoxic effects of non- functionalized and immune liposomes on HeLa cells (**A**), OVCAR-5 cells (**B**) and human fibroblasts (**C**). Only cells that received combined irradiation at 690 nm and 490 nm showed pronounced cell death (**A**). Differences between the two types of liposomes became more pronounced at lower concentrations of the conjugates, as shown here for OVCAR-5 cells (**B**). In fibroblasts no difference in cell elimination with immune or non-functionalized liposomes was observed (**C**). p values on the two linked columns from t-test analysis, indicate the statistically significant difference (p < 0.05) in cell viability between two samples.

Our previous studies demonstrated the ability of TuBB-9-FITC to kill proliferating cancer cells after irradiation at 490 nm. The irradiation of microinjected TuBB-9-FITC led to an inactivation of the Ki-67 protein or a binding partner and subsequent cell death^9^. To evaluate the efficiency of TuBB-9-FITC exerting their action after delivery by non-functionalized and immune liposomes, an *in vitro* cytotoxicity assay was performed. PCI with BPD and an irradiation at 690 nm was used to relocate the conjugates to the nucleoli. Figure 5 shows the cytotoxic effects of non- functionalized and immune liposomes on HeLa cells (A), OVCAR-5 cells (B) and human fibroblasts. Untreated HeLa cells and HeLa cells incubated only with BPD served as controls (Fig. 5A). Although the aim was to limit the damaging effects to the endosomal membrane, all the groups treated with BPD showed also a loss in cell viability of approximately 20–30% after 690 nm irradiation. This is caused by BPD phototoxicity. Without PCI, i.e. an irradiation only at 490 nm, cell viability decreased slightly for 10%. Only the combination of incubating with liposomes and BPD with irradiation at 690 nm and 490 nm, showed efficient cell killing with a reduction of cell viability to below 20%. Differences were observed between the non-targeted and immune-targeted liposomes. Immune liposome showed higher cell killing efficiency (cell viability of 11% ± 3%), compared to the group treated with non-functionalized liposome (17% ± 1%).

OVCAR-5 and fibroblast cells were additionally treated with non-functionalized and immune liposomes at different TuBB-9-FITC concentrations (Fig. 5B). At all investigated concentrations (50 nM, 25 nM and 12.5 nM) the EGFR over expressing OVCAR-5 cells showed higher cell killing efficiency with immune liposomes compared to non-functionalized liposome. Differences increased with lower concentrations and were significant at 5% level for 25 nM and 12.5 nM. As expected, in fibroblasts non-functionalized and immune liposomes showed no significant difference (Fig. 5C).

## Discussion

The development of molecular targeted therapies has to be based on detailed knowledge about the molecular mechanisms in cells and organisms, and sophisticated tools which allow interfering with these processes. In our study we established a dual target strategy, using EGFR on the cell surface and Ki-67 in the cell nucleus. Additional control is given by a light-activated intra-cellular release of the conjugates. Light inactivation of Ki-67 is a very effective mechanism to provoke cell death. Unlike many other Ki-67 antibodies the antibody TuBB-9 recognizes an active state of the protein, but shows no inhibitory effect when binding to Ki-67^9^,^14^. Light inactivation leads to photochemical crosslinking of the antibody and the target protein. Interacting partners of Ki-67 may also be part of the crosslinks and this may also be necessary for the apoptotic signal. Inactivation of Ki-67 has shown to be very selective against proliferating cells^10^. In many tumor tissues the proliferation index is higher than in normal tissue, but nevertheless the elimination of Ki-67 positive cells is per se not tumor selective. The spatial controlled application of light adds an additional level of selectivity, but for selectivity on cellular level we used the EGF-receptor as additional target. EGFR is overexpressed in many malignancies and the antibody Erbitux is approved as therapeutic antibody^32^.

Cell specific transport of drugs is an important part of new therapy approaches for personalized medicine. The conjugation of the EGFR antibody Erbitux to a maleimide-bearing lipid formed stable and reproducible liposomal constructs, which encapsulated the monoclonal Ki-67 antibody TuBB-9. Non-functionalized liposomes delivered comparable amounts of the Ki-67 antibody TuBB-9-FITC into the different cell lines HeLa, OVCAR-5, MCF-7 and human fibroblasts, regardless of their EGFR expression level. In contrast, our Erbitux conjugated immune liposomes were able to deliver TuBB-9-FITC at 2–4 times increased levels into cells depending on the cellular membrane EGFR expression status. Involvement of the EGF-receptor in the increased uptake was demonstrated by receptor blocking via prior incubation with Erbitux. Binding of liposomal Erbitux to the cell surface considerably enhances uptake of immune liposomes. Also in an EGFR not expressing cell line or after receptor blocking, a slightly better uptake of immune liposomes was observed. The antibody on the liposome surface seems to enhance to some extend the cellular endocytosis of the liposomes, regardless of the availability of the target protein on the cell surface. It is expected that this receptor independent mechanism reduces tumor selectivity.

Uptake in the cell alone is not sufficient to deliver the TuBB-9-FITC to the target structure, the nucleoli, since the content of the liposomes is trapped in endosomal structures inside the cells. Light irradiation at 690 nm, which activated the photosensitizer BPD, led to a rupture of endosomal membranes and released the TuBB-9-FITC antibody subsequently into the cytoplasm. 24 h and 48 h later the FITC fluorescence inside the nucleoli demonstrated successful binding of the antibody conjugates to Ki-67. The movement of the antibody from the cytoplasm to the nucleoli is presumably due to co-transport of the antibody with newly synthesized Ki-67 protein, or the antibody binds to Ki-67 during mitosis after breakdown of the nuclear envelope. At this point the second light irradiation at 490 nm photochemically cross-linked Ki-67 with interacting or close-by biomolecules^9^, resulting in cell death^10^. Immune liposomes caused higher cell death rates, compared to the non-functionalized liposomes. As we showed in earlier studies, cell death cannot reach 100%, since only approximately 90% of the monolayer cells are positive for Ki-67^30^.

In conclusion, we established a dual target, light-controlled delivery strategy for monoclonal antibodies with EGFR-targeting immune liposomes, light-controlled release from endosomes and light-mediated inactivation of Ki-67 as molecular switch to provoke efficient tumor cell elimination. Our approach combines the advantages of an effective and selective cellular transport with immune liposomes and photochemical internalization with the highly efficient cell killing by inactivating the nuclear protein Ki-67. The different steps of our strategy can also be applied for targeting and inactivation of other important membrane and intracellular molecules, and will be valuable tools for the further development of personalized medicine with molecular targets.

## Methods

### TuBB-9 antibody and FITC labeling

The monoclonal mouse antibody TuBB-9 was produced by hybridoma cells, kindly provided by the Leibnitz Research Center Borstel, Germany. Hybridoma cells were cultivated in bioreactor cell culture flasks (INTEGRA Biosciences, Switzerland). TuBB-9 was purified from the culture supernatant with protein G columns. For labeling with FITC (fluorescein 5(6)-isothiocyanate, Sigma, USA), TuBB-9 (1 mg/ml in sodium carbonate buffer at pH 9.3) was mixed with FITC (2.57 mM in DMSO) in a molar ratio of 20:1. The solution was incubated at room temperature on a shaker for 2 hours, and the TuBB-9-FITC conjugates were purified with a NAP-5 Sephadex column (GE Healthcare). After elution with TBS (10 mM Tris-HCl at pH 8.2, 150 mM NaCl), TuBB-9-FITC was concentrated with Microcon filter tubes (Millipore, USA) and resuspended in TBS (pH 7.4). In order to determine the labeling efficiency of FITC dye molecules per antibody the antibody concentration C_prot_ and FITC molecule number η were estimated by spectral measurements. For calculation the absorption at 280 nm and 495 nm was used in the empirical equations (1) and (2).


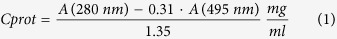



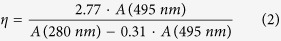


Calculation from absorption measurements showed, that between 1.5 and 2.4 molecules of FITC were bound to each antibody.

### Preparation and characterization of non-functionalized liposomes

Non-functionalized liposomes for encapsulating TuBB-9-FITC were prepared by dissolving the lipids DPPC, DOTAP, cholesterol, and PEG2000-DSPE (Avanti Polar Lipids, USA) in chloroform, in a ratio of 15:3:4:3. Chloroform was evaporated from the vial under a stream of nitrogen. The residual chloroform was removed by placing the vial in a desiccator under vacuum for 8 hours. 500 μl TuBB-9-FITC conjugates at a concentration of 5 mg/ml were then added at 50 °C, a temperature greater than the highest fluid-solid transition temperature (Tm: 48 °C) of the lipids in the mixture. The solution was incubated for 30 min at 50 °C, gently mixed and then incubated on ice. After 10 min the solution was incubated again for 10 min in the 50 °C water bath, these steps were repeated 6 times.

The resulting dispersion of multilamellar vesicles was extruded through a 100 nm polycarbonate membrane with a mini-extruder system (Avanti Polar Lipids, USA) to form unilamellar vesicles. A CL-4B Sephadex column was used to remove non-encapsulated conjugates. The resulting liposomes were analyzed by dynamic light scattering (DLS), and the concentration of encapsulated conjugates was determined by fluorescence and absorbance spectroscopy. The encapsulation efficiency of TuBB-9-FITC into liposomes [η (encapsulation)] was calculated with the help of an empirical equation (3).


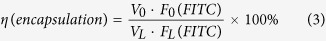


F_0_(FITC) and V_0_ indicate the measured fluorescence intensity of FITC at 490 nm and the volume of the TuBB-9-FITC solution added to the lipids during the preparation of liposomes. F_L_(FITC) and V_L_ indicate the fluorescence intensity of FITC at 490 nm and the volume of the prepared liposome solution encapsulating TuBB-9-FITC. According to over 10 preparations of liposomes, the encapsulation efficiency of TuBB-9-FITC into liposomes ranged between 7.6% and 11.2%. Liposomes were stored at 4 °C and used within the first two weeks after preparation.

### Preparation and characterization of immune liposomes

**Figure 6:**
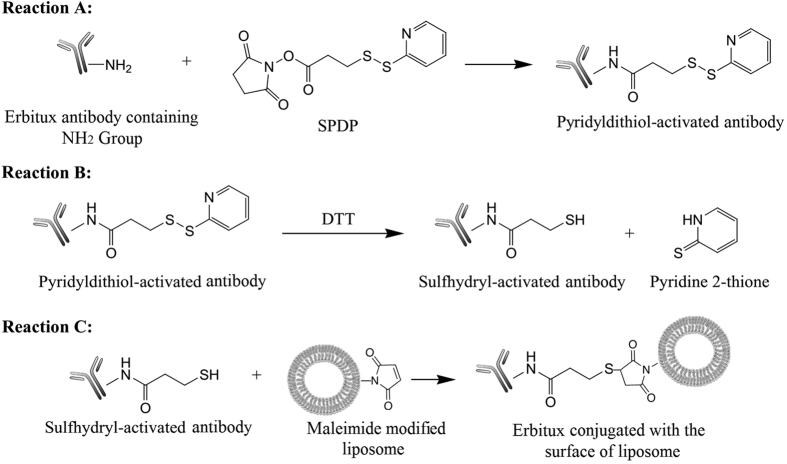
Chemical scheme for the synthesis of immune liposomes by conjugation of the EGFR antibody Erbitux to a maleimide-bearing lipid of the liposome. Pyridyldithiol groups were introduced to the antibody Erbitux with the help of a SPDP linker (Reaction **A**). The Pyridyldithiol-activated Erbitux was treated with dimethyl sulfoxide to break disulfide bonds (Reaction **B**). Finally, the Sulfhydryl-activated Erbitux reacts with maleimide modified liposomes (Reaction **C**).

Immune liposomes for the encapsulation of TuBB-9-FITC conjugates were prepared by referring to the method described by Ansell and colleagues (Ansell S. M., Harasym T. O.). First, maleimide groups were introduced into the liposome surface by adding 1% DSPE-PEG2000-Maleimide into the lipid film before liposome preparation as described in 2.2. The amount of lipids in the liposomes was determined by phosphate assay (Sigma, USA). Then we covalently conjugated the anti-EGFR antibody Erbitux (Merck, Germany) to the surface of the liposomes using the reactions showed in Fig. 6 with a linker molecule N-succinimidyl-3-(2-pyridyldithiol)propionate (SPDP; Pierce Chemical Co., USA). The Pyridyldithiol groups were introduced to Erbitux by the reaction of 0.9 mg Erbitux with 30 μL 1 mM SPDP in phosphate buffered saline (PBS, pH 7.6) in a ratio of 1:5 at room temperature for 30 min (Reaction A). The Pyridyldithiol-activated Erbitux was then purified on a NAP-5 mini-column (exclusion limit 5 kDa; GE Healthcare, USA) and eluated with acetate buffered saline (pH 4.4). The fractions containing Pyridyldithiol-activated Erbitux were treated with dimethyl sulfoxide (3.8 mg for 1 ml collected solution) for 20 min to break disulfide bonds (Reaction B). Then the solution was purified on a NAP-5 mini-column eluated with PBS (pH 7.0). The fractions containing thiol-activated Erbitux react with the prepared maleimide modified liposome solution (5 × 10^−4^ mol Erbitux per 1 mo1 of lipids) at room temperature for 16 h at pH 7.0 (Reaction C). After the reaction, a CL-4B Sephadex column was used to remove the non-conjugated Erbitux. The resulting immune liposomes were analyzed with the help of DLS, and the concentration of encapsulated conjugates was determined by fluorescence and absorbance measurements as described in the previous chapter. Absorbance was measured after dilution in 0.25% Triton-X100 to lyse the liposomes. The efficiency of the Erbitux conjugation to lipids was calculated with the help of equations (1) and (4).


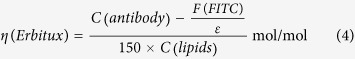


The empirical equation (1) allows for calculation of the antibody concentration C(antibody) (including Erbitux and encapsulated TuBB-9-FITC). Here, A(280 nm) and A(495 nm) indicate the absorption of the lysed liposome solution at 280 nm and 495 nm. Empirical equation (4) was used to estimate η(Erbitux), the molar amount of Erbitux per mol lipids in the liposomes. F(FITC) is the fluorescence intensity of FITC at 490 nm and ε the coefficient of the linear relationship between the FITC fluorescence intensity and the concentration. ε was determined with the help of the slope of a linear standard curve of the FITC fluorescence intensity from different known concentration of TuBB-9-FITC. C(lipids) is the amount of the total lipids in the immune liposome measured by a phosphate assay (Sigma Aldrich, USA). The concentration of Erbitux was calculated by subtracting the encapsulated TuBB-9-FITC from the total antibody concentration C(antibody). According to 6 preparations of immune liposomes, the liposomes contained about 6.0 × 10^−5^–10.7 × 10^−5^ mol Erbitux conjugated to 1 mol lipids. Since for the conjugation reaction, 75 μg (5.0 × 10^−10^ mol) Erbitux was added to react with the liposome containing 1 μmol (1 × 10^−6^ mol) lipids, the conjugation efficiency was between 12.0% and 21.3%. Liposomes were stored at 4 °C and used within the first two weeks after preparation.

### Cell culture

Human cervical cancer cell line HeLa, Human ovarian adenocarcinoma cell line OVCAR-5 and Human breast adenocarcinoma cell line MCF-7 were obtained from American Type Culture Collection (ATCC). Human dermal fibroblasts were obtained from the University Hospital Lübeck (UKSH, Lübeck, Germany). HeLa and MCF-7 cells were maintained in Dulbecco’s modified Eagle’s medium (DMEM low glucose, Sigma, USA) containing 10% fetal bovine serum (FBS gold, PAA, USA) and 1% Penicillin/Streptomycin (PAA, USA). OVCAR-5 cells were cultured in RPMI-1640 medium (Sigma, USA) containing 10% FBS and 1% Penicillin/Streptomycin. Fibroblast cells were maintained in DMEM medium (High glucose, Sigma, USA) containing 10% FBS and 1% Penicillin/Streptomycin.

### Cellular uptake of the liposomes

The amount of the internalized TuBB-9-FITC delivered by non-functionalized and immune liposomes into different cell lines was determined by the total fluorescence. 1 ml containing 1 × 10^5^ cells was plated in each well of a 24-well-plate. After four hours incubation with non-functionalized and immune liposomes containing 5 μg TuBB-9-FITC per well, cells were washed once with serum-free medium and twice with PBS. Cells were subsequently incubated with 100 ml RIPA lysis buffer for 10 minutes at 20 °C. The lysates were homogenized by pipetting up and down five times. The fluorescence signal was measured in 24-well plates with a multi-mode microplate reader (Spectramax M4, Molecular Devices, USA) in three independent experiments. The total protein amount in cell lysates was measured by protein assay. The internalized amount of TuBB-9-FITC was plotted as fluorescence amount per mg total protein. Error bars represent standard deviation.

### PCI irradiation and endosomal release

4 × 10^4^ HeLa cells/ml were seeded for 18–20 h in DMEM containing 100 nM BPD (benzoporphyrin derivative monoacid Ring A, Sigma, USA) in glass bottom imaging dishes. Cells were washed twice with PBS and incubated for four hours with non-functionalized and immune liposomes encapsulating 200 nM TuBB-9-FITC. Then the cells were washed once with serum-free DMEM and twice with PBS. Endosomal disruption by light irradiation was performed with a 690 nm LED with 18 mW/cm^2^ for 30 s. Fluorescence microscopy before and 48 h after irradiation was used to observe the localization of the TuBB-9-FITC conjugates (TE-Eclipse, Nikon, Japan).

### Cell viability assay

HeLa, OVCAR-5 and fibroblast cells were seeded at a density of 4 × 10^4^ cells/ml in 96-well plates, 100 nM BPD was added into each well, and incubated for 18 h. Then, cells were incubated for another 4 h with non-functionalized and immune liposomes encapsulating TuBB-9-FITC. For HeLa cells, a total amount of liposomes with 100 nM TuBB-9-FITC was used. For OVCAR-5 and fibroblast cells, liposomes with different TuBB-9-FITC concentrations (50 nM, 25 nM and 12.5 nM) were tested. After incubation, cells were washed twice with PBS and wells were divided into four groups: (1) no light irradiation, (2) irradiation with 690 nm laser for endosomal escape (18 mW/cm^2^, 30 s), (3) irradiation at 490 nm with a LED array for photo-inactivation of Ki-67 (60 mW/cm^2^, 3 min), (4) irradiation at 690 nm followed after 48 h by a second irradiation at 490 nm for endosomal escape and photo-inactivation of Ki-67. After 72 h, cells were washed twice with PBS and 100 μl MTT-solution (1 mg/ml in culture medium) was added per well. After an additional incubation for 1 h, culture medium was removed and the samples were incubated in 200 μl DMSO for 30 min on a shaker to dissolve the formazan crystals. Absorbance was measured on a microplate reader (Spectramax M5, Molecular Devices, USA) at 570 nm. The absorbance of treated samples compared to absorbance of untreated controls was used as a measure for cell viability. The viability data of different groups was expressed as mean value and standard deviation of in total eight measurements.

## Additional Information

**How to cite this article**: Wang, S. *et al*. A light-controlled switch after dual targeting of proliferating tumor cells via the membrane receptor EGFR and the nuclear protein Ki-67. *Sci. Rep.* **6**, 27032; doi: 10.1038/srep27032 (2016).

## Supplementary Material

Supplementary Information

## Figures and Tables

**Figure 1:**
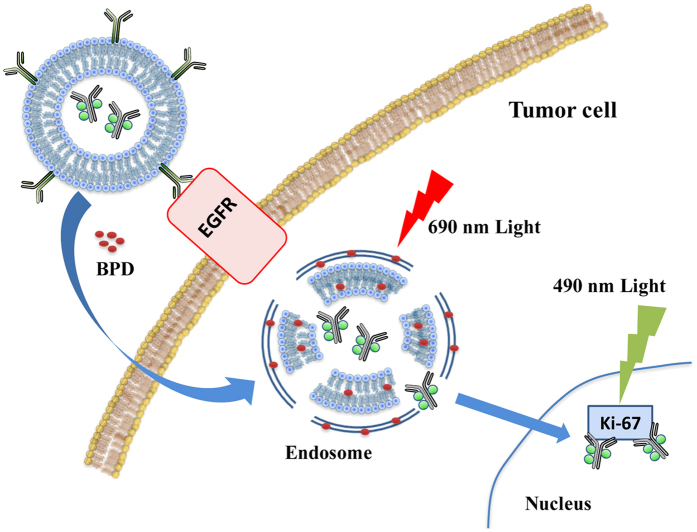
Scheme for the dually targeted strategy against the membrane protein *epidermal growth factor receptor* (EGFR) and the nuclear protein Ki-67.
