# Supplementary material for: A light-controlled switch after dual targeting of proliferating tumor cells via the membrane receptor EGFR and the nuclear protein Ki-67
Source: Sci Rep. 2016 Jun 1;6:27032. doi: 10.1038/srep27032 (PMC4887907; doi:10.1038/srep27032)
Supplement: Supplementary Information [file srep27032-s1.pdf]

Supplementary information for manuscript: SREP-16-07015A

Title:

A light-controlled switch after dual targeting of proliferating tumor cells via the membrane receptor EGFR and the nuclear protein Ki-67

Authors:

Sijia Wang, Gereon Hüttmann, Thomas Scholzen, Zhenxi Zhang, Alfred Vogel, Tayyaba Hasan, Ramtin Rahmanzadeh

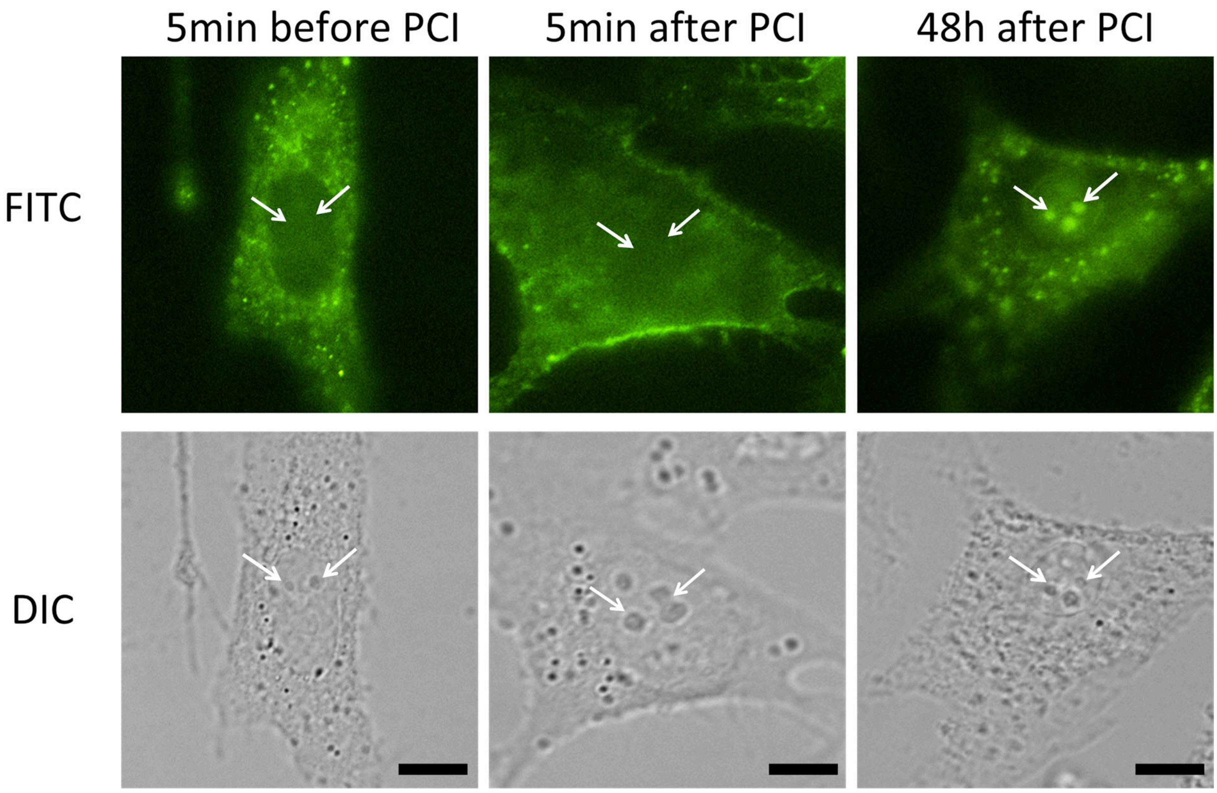

Supplementary figure S1: HeLa cells incubated with immune liposomes encapsulating TuBB-9-FITC. Before PCI irradiation (690 nm) the FITC fluorescence (upper panels) is located in endosomal structures and not in the nucleoli (arrows; lower panels show differential interference contrast, DIC). 5 min after irradiation FITC fluorescence is spread through the cytoplasm indicating the opening of the endosomes. 48 h after PCI irradiation the fluorescence of TuBB-9-FITC can be detected inside the nucleoli, indicating the binding to Ki-67. Size bars: 5 µm.
